# The Prognostic Value of AT-Rich Interaction Domain (ARID) Family Members in Patients with Hepatocellular Carcinoma

**DOI:** 10.1155/2022/1150390

**Published:** 2022-08-18

**Authors:** Siyi Li, Zhulin Wu, Qiuyue Li, Qiting Liang, Hengli Zhou, Yafei Shi, Rong Zhang, Huafeng Pan

**Affiliations:** ^1^Joint Laboratory for Translational Cancer Research of Chinese Medicine of the Ministry of Education of the People's Republic of China, Guangzhou 510405, China; ^2^International Institute for Translational Chinese Medicine, Guangzhou University of Chinese Medicine, Guangzhou 510405, China; ^3^The Fourth Clinical Medical College of Guangzhou University of Chinese Medicine, Shenzhen 518033, Guangdong, China; ^4^Science and Technology Innovation Center, Guangzhou University of Chinese Medicine, Guangzhou 510405, China; ^5^Institute of Clinical Pharmacology, Guangzhou University of Chinese Medicine, Guangzhou 510405, China; ^6^Basic Medical College of Guangzhou University of Chinese Medicine, Guangzhou 510405, China

## Abstract

**Objective:**

Hepatocellular carcinoma (HCC) is one of the most lethal malignancies with a poor prognosis. The AT-rich interaction domain (ARID) family plays an essential regulatory role in the pathogenesis and progression of cancers. This study aims to evaluate the prognostic value and clinical significance of human ARID family genes in HCC.

**Methods:**

ONCOMINE and The Cancer Genome Atlas (TCGA) databases were employed to retrieve ARIDs expression profile and clinicopathological information of HCC. Kaplan–Meier plotter and MethSurv were applied to the survival analysis of patients with HCC. CBioPortal was used to analyze genetic mutations of ARIDs. Gene Expression Profiling Interactive Analysis (GEPIA) and Metascape were used to perform hub gene identification and functional enrichment.

**Results:**

Expression levels of 11 ARIDs were upregulated in HCC, and 2 ARIDs were downregulated. Also, 4 ARIDs and 5 ARIDs were correlated with pathologic stages and histologic grades, respectively. Furthermore, higher expression of ARID1A, ARID1B, ARID2, ARID3A, ARID3B, ARID5B, KDM5A, KDM5B, KDM5C, and JARID2 was remarkably correlated with worse overall survival of patients with HCC, and the high ARID3C/KDM5D expression was related to longer overall survival. Multivariate Cox analysis indicated that ARID3A, KDM5C, and KDM5D were independent risk factors for HCC prognosis. Moreover, ARIDs mutations and 127 CpGs methylation in all ARIDs were observed to be significantly associated with the prognosis of HCC patients. Besides, our data showed that ARIDs could regulate tumor-related pathways and distinct immune cells in the HCC microenvironment.

**Conclusions:**

ARIDs present the potential prognostic value for HCC. Our findings suggest that ARID3A, KDM5C, and KDM5D may be the prognostic biomarkers for patients with HCC.

## 1. Introduction

Liver cancer is the leading cause of mortality among malignancies according to the statistics from the GLOBOCAN database [1]. Hepatocellular carcinoma (HCC), the most prevalent subtype of liver cancer, accounts for 75%–85% of all liver cancer cases worldwide in 2020 [2]. In addition, the five-year survival rate of HCC is approximately 18.1% due to lack of information for early diagnosis and limited therapeutic strategy [3]. Most of the patients with HCC are at stages of an interim or advanced period when they are diagnosed, who are not applicable for surgical treatment [4]. Some drugs, such as regorafenib, nivolumab, and lenvatinib, have been developed for HCC treatment [5]. However, drug resistance and adverse effects restrain the overall therapeutic efficacy of these drugs [6]. Therefore, it is urgently needed to find effective markers for detection, diagnosis, and prognosis of patients with HCC in the early stage.

Human AT-rich interaction domain (ARID) family composes of 15 members, including ARID1A, ARID1B, ARID2, AIRD3A, ARID3B, ARID3C, ARID4A, ARID4B, ARID5A, ARID5B, KDM5A (JARID1A), KDM5B (JARID1B), KDM5C (JARID1C), KDM5D (JARID1D), and JARID2. According to sequence identity among individual members, the ARID family was classified into 7 subfamilies, containing ARID1, ARID2, ARID3, ARID4, ARID5, KDM5, and JARID2. The ARID family genes have the ability of DNA binding, which may participate in the modification of chromatin structure and play a positive or negative role in regulating the transcription of target genes [7]. As transcriptional regulators, ARIDs have been found to be associated with cell growth, differentiation, and development, which is also closely associated with cancers [8]. For instance, a previous study has found many meaningful ARID family biomarkers in breast cancer [9]. ARID family is associated with the immune infiltrate and tumor microenvironment of digestive cancer [10]. In addition, ARID1A silencing promotes epithelial-mesenchymal transition and enhances the sensitivity of pancreatic tumor cells to NVP-AUY922, which is a promising biomarker for the identification of pancreatic ductal adenocarcinomas [11]. ARID family members are also identified as novel biomarkers for immune checkpoint inhibitor therapy in malignancies by pan-cancer analysis [12]. Nevertheless, the prognostic value of ARIDs in HCC has not been thoroughly studied.

In the current study, the expression, mutation, and methylation of different ARIDs were analyzed, and their correlation with clinicopathological parameters and survival of HCC patients was unveiled. Besides, the predicted functions and immune regulating roles of ARIDs were also analyzed.

## 2. Methods

### 2.1. Gene Expression Analysis of ARIDs

First of all, the differential expression of ARIDs between HCC and normal tissues was analyzed using the ONCOMINE (https://www.oncomine.org) database [13]. We followed the methods of Wu et al. with some modifications [14]. Screening criteria were as follows: cancer type = liver cancer; gene = ARID family members; data type = mRNA; analysis type = cancer versus normal analysis; threshold values: *p* value < 0.05, fold change > 1.5, and gene rank = top 10%. By default, the difference between normal and HCC tissue groups was analyzed using the *t*-test. Subsequently, differences in ARID family gene expression between HCC and normal samples were compared. Moreover, The Cancer Genome Atlas (TCGA, https://tcga-data.nci.nih.gov/tcga/) [15] dataset was also used to analyze the mRNA expression of 15 ARID family members in HCC tissues, and result visualization was performed with “ggplot2” package in R software (version 3.6.3). In addition, the association between ARID family members and clinicopathologic parameters (pathologic stage and histologic grade) was evaluated using the TCGA HCC dataset. Clinical information for pathological stages I and II (early stage) and stages III and IV (advanced stage), and histologic grades of patients with HCC were obtained from the TCGA database. HCC patients who lacked information on pathologic stage or histologic grade were excluded, and results were visualized with violin plots using the “ggplot2” package in R software.

### 2.2. Prognostic Value of ARID Family Genes

Association between the ARIDs expression and HCC prognosis was evaluated by the online software Kaplan–Meier (KM) plotter (https://www.kmplot.com) [16, 17]. Patients with HCC in TCGA dataset were divided into two groups based on the optimal cutoff value of ARIDs expression, and overall survival (OS) was selected as the outcome. To evaluate the independent prognostic factors of ARIDs, univariate and multivariate Cox analyses were conducted for both ARIDs and clinicopathological data (age, gender, stage, and *T* stage) using the TCGA HCC dataset, and factors with *p* value < 0.1 were selected for multivariate analysis. Additionally, MethSurv, an online tool for methylation visualization (https://biit.cs.ut.ee/methsurv/) [18] was applied to perform DNA methylation-based survival analysis of ARIDs using the TCGA dataset.

### 2.3. Genetic Mutations in ARID Family Genes

Mutations of ARID family members in HCC were analyzed via the cBioPortal tool (https://cbioportal.org) [19] by referring to a previous study [20]. Furthermore, KM plots were utilized to evaluate the correlation between genetic mutations in ARIDs and the survival time of HCC patients with cBioPortal. In cBioPortal analysis, HCC patients were classified into the altered and unaltered groups. OS, progression-free survival (PFS), and disease-free survival (DFS) were all considered as endpoints.

### 2.4. Functional Enrichment Analyses

Hub genes screening and functional enrichment analyses were carried out according to the previously reported methods [21]. Before functional enrichment analysis, the top 10 similar genes of each ARID family member were acquired using the Gene Expression Profiling Interactive Analysis (GEPIA, https://gepia.cancer-pku.cn/) [22]. Functions of ARID family genes were identified using the Metascape database (https://metascape.org) [23]. Additionally, Gene Ontology (GO) analysis was used to determine the biological functions of these genes based on three categories, including cellular components, molecular functions, and biological processes. Kyoto Encyclopedia of Genes and Genomes (KEGG) analysis was applied to identify signaling pathways related to ARIDs and their similar genes. Moreover, protein-protein interaction (PPI) network establishment and independent functional enrichment analyses of Molecular Complex Detection (MCODE) components were carried out in Metascape.

### 2.5. Immune Infiltrates Correlation Analysis

Tumor Immune Estimation Resource (TIMER, https://cistrome.shinyapps.io/timer/), an online tool for comprehensive analysis of tumor-infiltrating immune cells [24], was used to assess the correlation between ARID family genes and immune infiltrates. We followed the methods of Qin et al. to perform the immune infiltrates correlation analysis [25]. The relationships of ARIDs expression with tumor-infiltrating immune cells (B cell, CD4^+^ T cell, CD8^+^ cell, macrophage, neutrophil, and dendritic cell) were identified by purity-corrected partial Spearman's correlation (partial-cor) and statistical significance, and the results were shown in scatterplots provided by TIMER.

### 2.6. Real-Time Quantitative Reverse Transcription Polymerase Chain Reaction (RT-qPCR)

Total RNA was extracted from LO2 and HepG2 cells using TRIzol reagent. We synthesized cDNA from total RNA using the One Step PrimeScript RT-PCR Kit. Next, RT-qPCR was conducted and analyzed using the ChamQTM Universal SYBR qPCR Master Mix (Vazyme, Nanjing, China) and the ABI 7500 sequence detection system. The cycling condition for RT-qPCR was 95°C for 3 min, 45 cycles of 95°C for 5 s, 60°C for 30 s, and 72°C for 10 s. Each experiment was conducted in triplicate. Primer sequences are listed in Table 1.

### 2.7. Statistical Methods

In the TCGA HCC dataset, differences between normal and HCC tissue groups were analyzed by *t*-test or Mann–Whitney *U* test. ANOVA test or Kruskal–Wallis test was used for multiple comparisons. Correlations among ARIDs were estimated using the Spearman test. Survival curves were plotted by the KM method, with hazard ratios (HRs) with 95% confidence intervals (CIs) and log-rank *p* values. In this paper, statistical analyses were conducted using the *R* software (version 3.6.3) or online databases. *p* value < 0.05 are statistically significant.

## 3. Results

### 3.1. Gene Expression Analysis of ARIDs

**Figure 1:**
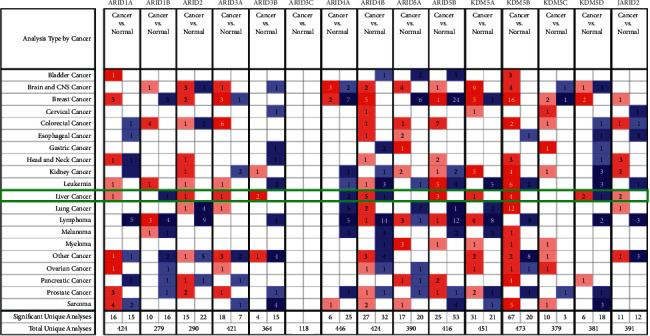
The mRNA expression of AT-rich interaction domain (ARID) family members in multiple cancers and hepatocellular carcinoma (HCC) based on ONCOMINE. Blue and red colors, respectively, indicate low and high expression. The number in each box represents the number of datasets that fulfill the criteria for this study.

**Figure 2:**
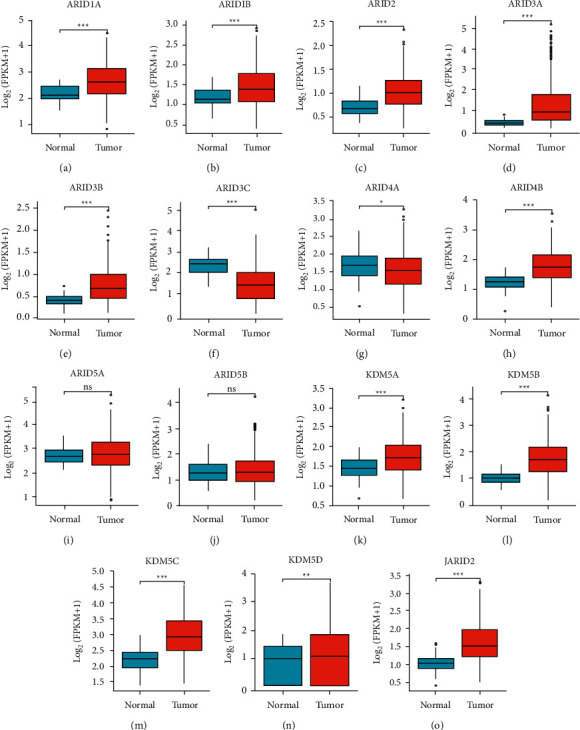
Expression of 15 ARID family members in HCC and normal tissues. (a-o) The mRNA expression levels of ARIDs were increased in HCC compared with normal tissues based on The Cancer Genome Atlas (TCGA) dataset. ns represents *p* ≥ 0.05, ^*∗*^ represents *p* < 0.05, ^*∗∗*^ represents *p* < 0.01, and ^*∗∗∗*^ represents *p* < 0.001.

As displayed in Figure 1, 15 ARIDs were identified in distinct cancers, containing breast cancer, liver cancer, brain cancer, and central nervous system (CNS) cancer. The expression levels of ARID1A, ARID2, ARID3A, ARID3B, ARID4B, ARID5B, KDM5A, KDM5B, KDM5D, and JARID2 were upregulated, and expression levels of ARID1B, ARID4A, ARID4B, and KDM5D were downregulated in all types of liver cancers (including hepatocellular adenoma, liver cell dysplasia, HCC, and cirrhosis). Furthermore, data from four datasets [26–28] in ONCOMINE demonstrated that ARID1A, ARID2, ARID3A, ARID4B, KDM5A, KDM5B, and JARID2 were markedly upregulated in HCC than that in normal groups (Table 2). Furthermore, based on the TCGA dataset, mRNA expression of ARID1A, ARID1B, ARID2, ARID3A, ARID3B, ARID4B, KDM5A, KDM5B, KDM5C, KDM5D, and JARID2 was remarkably overexpressed, and ARID3C and ARID4A were underexpressed in HCC (Figure 2). In addition, no significant difference was found regarding ARID5A or ARID5B mRNA expression in the TCGA HCC dataset. Subsequently, obvious correlations among ARIDs were discovered using the Spearman test (Supplementary Figure 1).

### 3.2. Correlations between Clinical Features and ARIDs Expression in HCC

**Figure 3:**
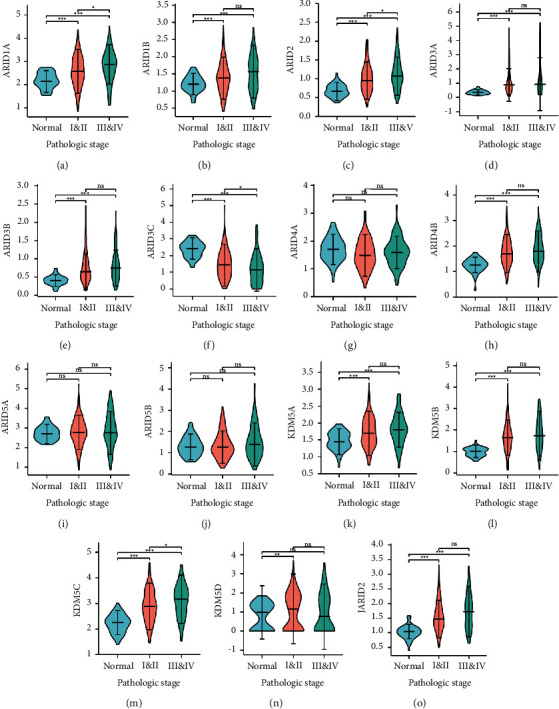
The relationship between the expression of different ARIDs and pathologic stages (TCGA dataset). (a–o) Violin plots showing the expression of the 15 ARID family genes with the pathologic stages (normal, stage I/II, and stageIII/IV). ARID1A, ARID2, and KDM5C were highly expressed in advanced stage (a, c, m) and lower mRNA expression of ARID3C was detected in the early stage (f). ^*∗*^ indicates *p* < 0.05, ^*∗∗*^ indicates *p* < 0.01, ^*∗∗∗*^ indicates *p* < 0.001, and ns represents *p* ≥ 0.05.

**Figure 4:**
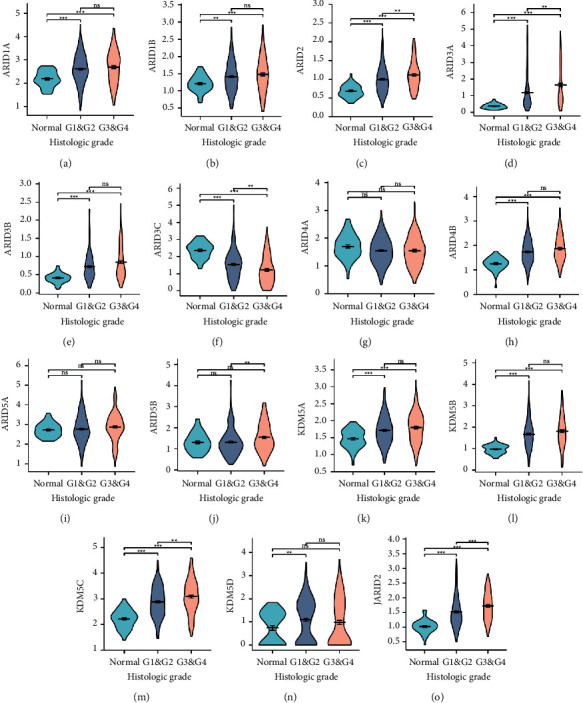
Association between the expression of distinct ARIDs and histologic grades. (a–o) Violin plots showing the expression of the 15 ARID family genes with grades (normal, G1/2, and G3/4). ARID2, ARID3A, ARID3B, ARID5B, KDM5C, and JARID2 were highly expressed in G3/4 (c, d, e, j, m, o), and lower mRNA expression of ARID3C was found in G1/2 (f). ^*∗∗*^ indicates *p* < 0.01, ^*∗∗∗*^ indicates *p* < 0.01, and ns represents *p* > 0.05.

To identify correlations of ARIDs expression with clinicopathological factors, the clinical data of pathologic stage and histologic grade were analyzed based on the TCGA database. Results showed that higher expression levels of ARID1A, ARID2, and KDM5C were closely associated with advanced pathologic stages of patients with HCC (Figures 3(a), 3(c), and 3(m)). Increased ARID3C expression was related to the late pathologic stage (Figure 3(f)). Moreover, violin plots displayed that the expression levels of ARID2, ARID3A, ARID3C, ARID5B, KDM5C, and JARID2 were remarkably related to grades (Figures 4(a)–4(o). Higher expression levels of ARID2, ARID3A, ARID5B, KDM5C, and JARID2 tended to be correlated to higher histologic grade (Figures 4(c), 4(d), 4(j), 4(m), and 4(o)). ARID3C expression was negatively associated with the histologic grade of patients with HCC (Figure 4(f)).

### 3.3. Prognostic Value of ARIDs Members in HCC

**Figure 5:**
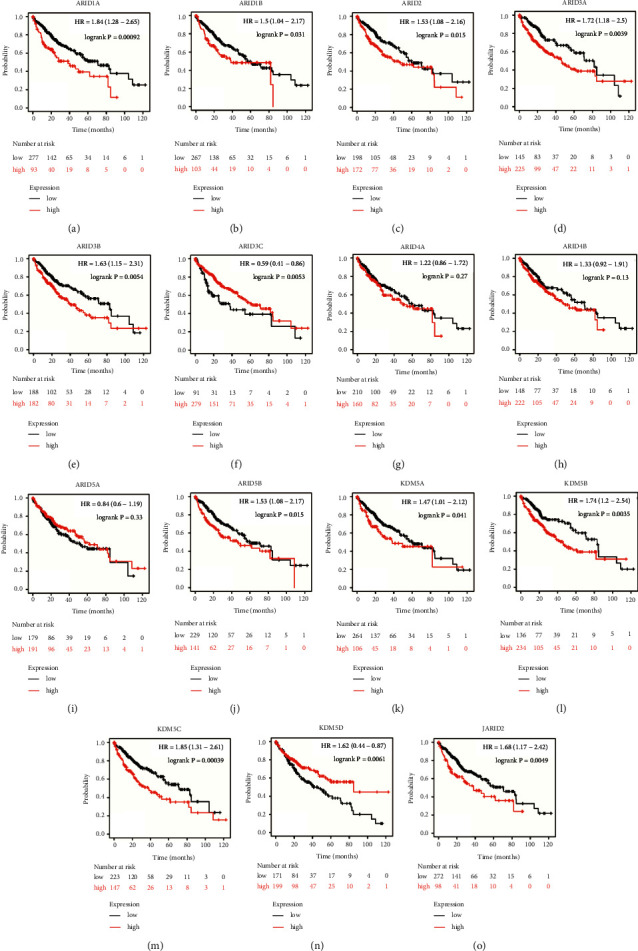
The prognostic value of ARID family genes in HCC patients (Kaplan–Meier plotter). (a–o) The Kaplan–Meier survival curves comparing HCC patients with high (red) and low (black) expressions of ARIDs were plotted. High expression levels of ARID1A (a), ARID1B (b), ARID2 (c), ARID3A (d), ARID3B (e), ARID5B (j), KDM5A (k), KDM5B (l), KDM5C (m), and JARID2 (o) were significantly correlated with shorter overall survival (OS) in HCC patients. High expression levels of ARID3C (f) and KDM5D (n) were remarkably associated with better OS. Others showed no correlation with OS time.

The prognostic value of ARID family members in HCC was identified using a KM plotter. KM plot presented that high expression levels of 10 ARIDs, including ARID1A, ARID1B, ARID2, ARID3A, ARID3B, ARID5B, KDM5A, KDM5B, KDM5C, and JARID2, were associated with the shorter OS of all patients with HCC (Figures 5(a)–5(d), 5(e), and 5(j)–5(o)). Conversely, high mRNA expression levels of ARID3C and KDM5D were obviously associated with better OS time (Figures 5(f) and 5(n)). In addition, the association between ARID4A/ARID5B/ARID5A expression and OS in patients with HCC did not show statistical significance (Figures 5(g)–5(i)). Also, the prognostic value of CpGs was analyzed using MethSurv, and a total of 127 –CpGs in ARIDs were found to be associated with the prognosis of patients with HCC (Supplementary Table 1). In univariate Cox analysis, pathologic stage, *T* stage, and some ARIDs (including ARID2, ARID3A, ARID3B, KDM5B, KDM5C, and KDM5D) were correlated to OS of HCC patients (Supplementary Tables 2–16). Furthermore, multivariate Cox analysis indicated that high expression levels of ARID3A, KDM5C, and KDM5D were independently correlated with worse OS in patients with HCC.

### 3.4. Genetic Mutations of ARIDs in Patients with HCC

**Figure 6:**
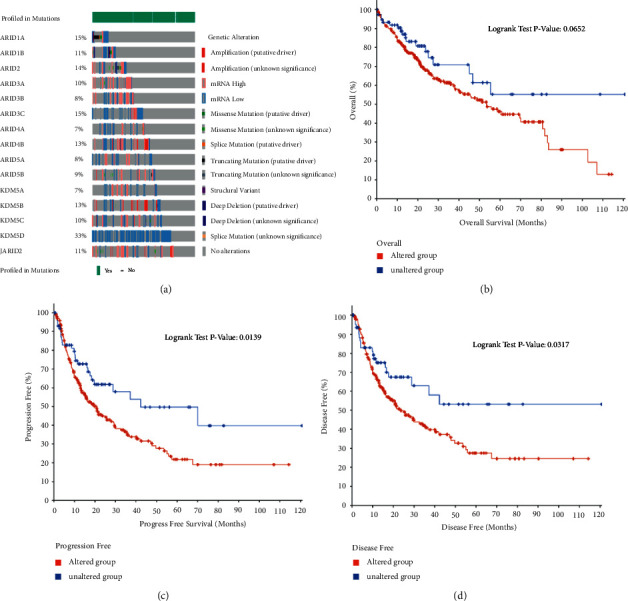
Alteration frequency of ARID family genes and their correlation with prognosis of HCC patients (cBioPortal database). (a) The ARIDs mutation rate was 78.69% (288/366) in patients with clear cell renal cell carcinoma (ccRCC). The top three highest alteration rates in ARID family genes were KDM5D (33%), ARID3C (15%), and ARID1A (15%), respectively. (b) Genetic alterations in ARIDs showed no correlation with OS time. (c) Genetic alterations in ARID family genes were remarkably related to progression-free survival (PFS) of ccRCC patients (*p* < 0.05). (d) Genetic alterations in ARID family genes were significantly related to disease-free survival (DFS) of HCC patients (*p* < 0.05).

The genetic alterations of ARID family members in the TCGA dataset are displayed in Figure 6(a). Among 366 patients with HCC, 288 patients have genetic mutations of ARID family genes, with 78.69% mutation rate of ARIDs. The percentages of genetic mutations of ARIDs in HCC ranged from 7% to 33%, and the top 3 alteration rates in ARID family genes were KDM5D (33%), ARID3C (15%), and ARID1A (15%). The KM survival plot of OS indicated that the mutations of ARIDs were related to the poor prognosis of patients with HCC (Figure 6(b)). As displayed in Figures 6(c) and 6(d), genetic alterations in ARID family genes were related to worse PFS (*p*=0.0139) and DFS (*p*=0.0317) of patients with HCC. Collectively, genetic mutations of ARID family members may remarkably influence the survival of patients with HCC.

### 3.5. Functional Analyses of ARIDs in HCC

**Figure 7:**
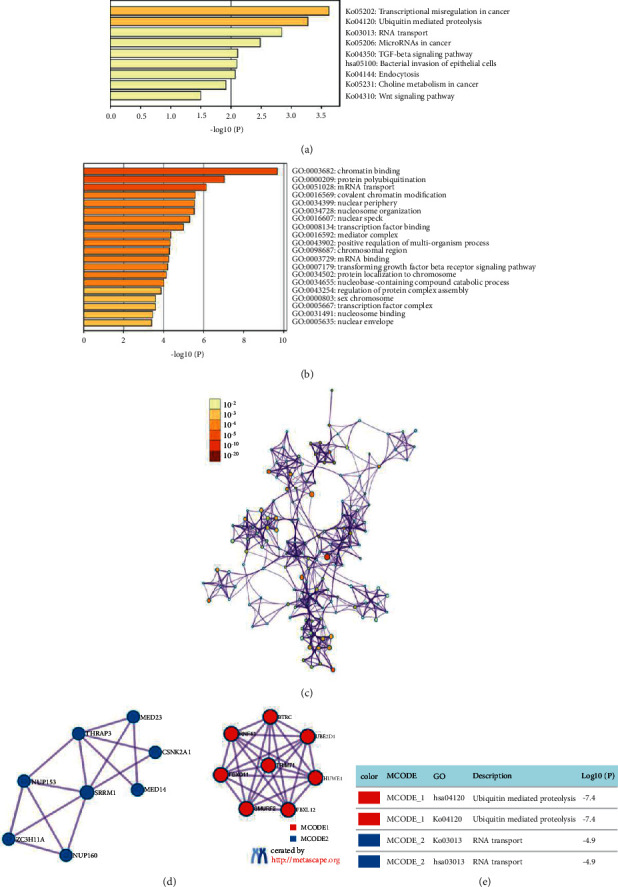
Functional enrichment analysis of ARID family members and their similar genes in HCC patients (GEPIA and Metascape). (a) Barplot of KEGG pathways colored by the value of -log10 (*p* value). (b) Barplot of GO enriched terms colored by -log10 (*p* value). (c) The network of GO enriched terms. (d) Two key MCODE components form the PPI network. (e) Independent functional enrichment analysis of MCODE components.

The top 10 similar genes of each ARID family gene were collected from the GEPIA database (Supplementary Table 17). The functions of ARIDs were predicted using Metascape with the thresholds of *p* -value < 0.05, min overlap = 3, and min enrichment = 3. Following the removal of the duplicate genes, 140 genes were collected, including 15 ARID family genes and 125 similar genes. As shown in Figure 7(a), KEGG pathways, including transcriptional misregulation in cancer, mircoRNAs in cancer, ubiquitin-mediated proteolysis, transforming growth factor (TGF)-beta signaling pathway, bacterial invasion of epithelial cells, endocytosis, choline metabolism in cancer, and Wnt signaling, were correlated with the biological functions of ARIDs in HCC. Moreover, the enriched GO terms of these genes were classified into three groups: 9 biological processes, 4 molecular functions, and 7 cellular component terms. As displayed in Figures 7(b) and 7(c), these genes were mainly related to chromatin binding, protein polyubiquitination, mRNA transport, and covalent chromatin modification. Additionally, the two most crucial MCODE components were identified from PPI analysis. Results showed that biological functions were mostly related to ubiquitin-mediated proteolysis (MCODE 1), and RNA transport (MCODE (2) (Figure 7(e)).

### 3.6. Immune Regulating Roles of ARIDs

**Figure 8:**
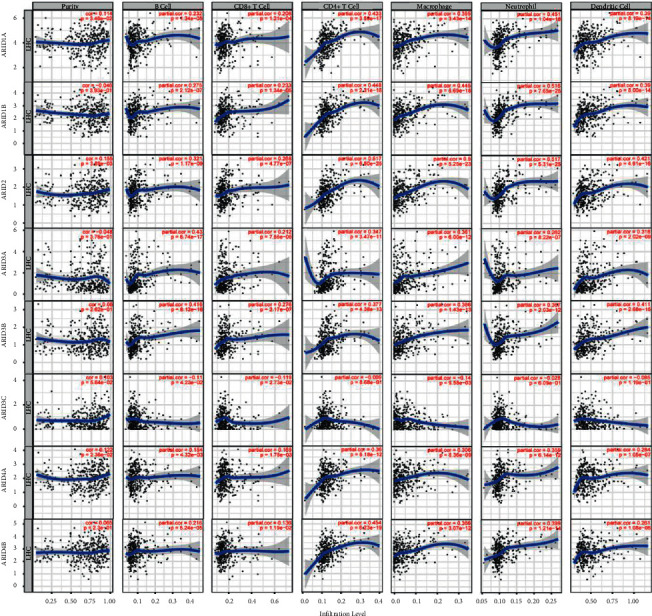
Relationship between the expression of ARID1A, ARID1B, ARID2, ARID3A, ARID3B, ARID3C, ARID4A, and ARID4B and immune infiltration level by TIMER in HCC. The left-most panels show tumor purity, and the associations of tumor-infiltrating immune cells and these genes in HCC were displayed.

**Figure 9:**
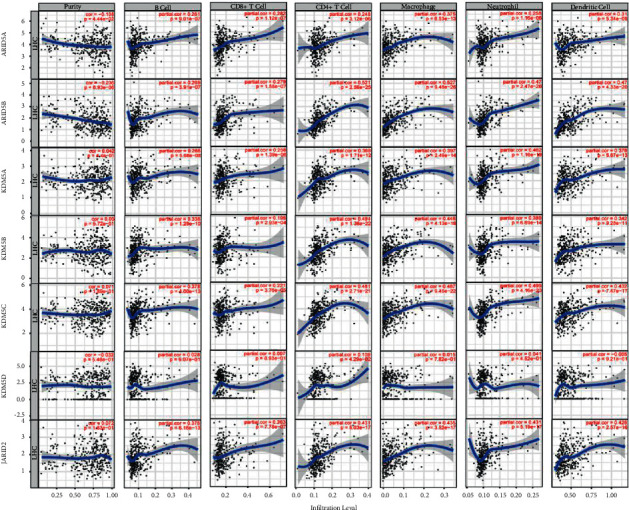
Relationship between the expression of ARID5A, ARID5B, KDM5A, KDM5B, KDM5C, KDM5D, and JARID2 and immune infiltration level by TIMER in HCC. The left-most panels show tumor purity, and the associations of tumor-infiltrating immune cells and these 7 genes in HCC were shown.

As shown in Figures 8 and Figure 9, results from the TIMER database showed that the expression of ARID1A, ARID2, and ARID4A was positively related to tumor purity (*p* < 0.05), indicating that these three genes were lowly expressed in the HCC immune microenvironment. Conversely, a negative correlation between ARID5A/ARID5B and tumor purity was found. Moreover, except for ARID3C and KDM5D, other ARID family members were significantly associated with all six types of immune cells (B cell, CD4^+^ T cell, CD8^+^ T cell, macrophage, neutrophil, and dendritic cell) in HCC microenvironment.

## 4. Discussion

Hepatocellular carcinoma (HCC) is a common malignancy belonging to liver cancer with high mortality. Patients with HCC are often diagnosed at the middle or advanced stage with a poor prognosis. Therefore, the identification of novel prognostic and diagnostic markers is urgently needed. As transcriptional regulators, ARIDs regulate cellular growth, differentiation, and development in a variety of cancers [29]. Zhang et al. identified the prognostic value of ARIDs and provided insight to explore the ARID-targeting regents for breast cancer treatment [9]. Sun et al. revealed the association between ARIDs, prognosis, and the tumor microenvironment in HCC, indicating that ARIDs are prospective therapeutic targets for HCC [30]. Growing evidence demonstrates that ARID family genes act as tumor promoters or suppressors to regulate the occurrence and development of cancers [8]. A pan-cancer analysis for ARID family members exhibits that ARIDs are novel biomarkers for immune checkpoint inhibitor therapy [12].

ARID1A is highly expressed in primary HCC, and the overexpression of ARID1A could accelerate tumor initiation [31]. Consistently, our study found that ARID1A was highly expressed in HCC and associated with pathologic stage and OS time. However, loss of homozygous or heterozygous ARID1A in HCC may accelerate progression [31]. Therefore, ARID1A plays a context-dependent function in tumor inhibition and carcinogenesis in HCC. Hu et al. reported that ARID1A deficiency could increase tumor mutation burden, upregulate the programmed cell death ligand 1 (PD-L1) expression, and modulate the immune microenvironment [32], which supports our findings. Interestingly, growth-promoting effect was observed for the knockdown of ARID1B in HCC [33], which is contrary to our results. The abnormality of ARID1B may destroy the function of Switch/sucrose-nonfermentable (SWI/SNF) complex in the regulation of gene expression and antioncogenic and oncogenic pathways, which are related to the carcinogenesis of HCC [34]. Additional studies are still needed to explore the modulatory pathways of ARID1A and ARID1B.

Our data showed that ARID2 was highly expressed in HCC and associated with clinicopathological factors and poor prognosis. ARID2 is an important tumor suppressor in HCC, but recent genomic studies have found frequent mutations of ARID2 in HCC [35]. The nucleotide change of ARID2 is also considered to be the driver of HCC [36]. Furthermore, inactivating mutations of ARID2 in three main types of HCC, including hepatitis C virus (HCV)-related, hepatitis B virus (HBV)-related, and alcohol-related HCC were found [37]. Combined with previous studies, our discoveries further confirmed that ARID2 may be a promising prognostic and therapeutic target for HCC.

ARID3 subfamily, containing ARID3A, ARID3B, and ARID3C, regulates gene expression by binding to specific DNA common sites, playing a key role in transcriptional regulation [38]. Our study found that ARID3A and ARID3B were overexpressed in HCC and associated with poor OS. In particular, ARID3A was an independent prognostic factor for predicting OS of patients with HCC, and HCC patients with advanced grades tended to have higher mRNA expression of ARID3A. ARID3A is reportedly involved in a variety of biological processes and maybe correlated with tumorigenesis. Also, overexpression of ARID3A could potentiate cancer cell proliferation, migration, and invasion [39]. In addition, previous research has shown that ARID3A and ARID3B jointly regulate gene expression in B-cells and cancers, regulate stem cell-related genes, and promote the phenotype of tumor stem cells [38]. Additionally, ARID3C was observed to be a protective prognostic factor for HCC. ARID3C has been identified as one of the main downstream targets of *β*-catenin, and ARID3C knockdown could inhibit cell proliferation of ovarian cancer [40]. Studies on ARID3C in HCC are still limited, but our data provided some light into this field.

ARID4A plays a dual role in cancer progression. In patients with prostate cancer, the down-regulation of ARID4A promotes tumor progression [41]. ARID4A is a tumor suppressor in breast cancer, the expression of which indicates better OS in patients with breast cancer [9]. However, our data demonstrated that ARID4A was not significantly correlated with OS or clinicopathological in HCC. Moreover, it has been reported that the expression of ARID4B in HCC tissues was upregulated compared to that in adjacent normal liver tissues, which is an independent prognostic factor for predicting OS and DFS of patients with HCC [42]. Although we also found that ARID4B was upregulated in HCC, OS is not significantly different between the low and high ARID4B expression groups. Thus, research with a larger sample size is warranted.

ARID5 subfamily includes ARID5A and ARID5B. The research found that ARID5A regulates the inflammatory process, low expression of which is correlated with poor prognosis of lung cancer patients [43]. ARID5A is also a prognostic biomarker for glioma, which is correlated with immune infiltration [44]. ARID5B is associated with histone deacetylase-1, thereby affecting cell proliferation and differentiation [45]. In our study, KM survival curves displayed that high ARID5B expression was associated with worse OS time, but the association between ARID5A and OS of patients with HCC did not reach statistical significance.

Our results revealed that KDM5A, KDM5B, KDM5C, and KDM5D were upregulated in HCC tissues. A previous study indicates that KDM5A depletion leads to reduced cell migration, invasion, and proliferation of HCC, which may promote angiogenesis by activating the PI3K/AKT pathway [46]. Also, KDM5B is highly expressed in HCC, promoting the occurrence of HCC by regulating the YTHDF3/ITGA6 axis [47]. Meanwhile, we found that high expression levels of KDM5A, KDM5B, and KDM5C were associated with shorter OS, and high expression of KDM5D was correlated with longer OS time. In particular, patients with HCC at an advanced stage or grade have higher KDM5C mRNA expression. Evidence has demonstrated that the overexpression of KDM5C could predict a poor prognosis of HCC patients undergoing radical resection and promotes the HCC cell invasion, metastasis, and epithelial-mesenchymal transition, suggesting that KDM5C may be a potential therapeutic target for HCC [48]. Multivariate Cox analysis showed that KDM5C or KDM5D was an independent prognostic factor for the prediction of OS. KDM5D regulates the epithelial-mesenchymal transformation and metastasis of gastric cancer, which is a novel target for cancer treatment [49]. Besides, JARID2 was elevated in HCC, high expression of which was remarkably related to late grade and worse prognosis. Preceding research has shown that the expression of JARID2 in HCC is significantly upregulated, which is closely associated with the metastasis of HCC. JARID2 could also promote invasion, metastasis, and epithelial-mesenchymal transition of HCC cells via the PTEN/AKT pathway [50].

The frequent mutations of ARIDs have been well-established using the cBioPortal database, which is supported by previous studies [8, 33]. ARIDs mutations are correlated with poor prognosis in patients with HCC. CpGs methylation in all ARIDs may serve as prognostic markers for HCC patients. To further study the potential mechanism of ARIDs in HCC, functional analysis was performed, and the results showed that ARIDs and their similar genes were involved in the signaling pathways related to inflammatory responses and cancer. Evidence showed that inefficient proteolysis can lead to the dysregulated cell cycle transition, which eventually results in tumorigenesis. Therefore, ubiquitin ligases related to cell cycle regulation are expected to become a therapeutic strategy for cancer [51]. Additionally, abnormal activation of the Wnt/*β*-catenin signal plays an important role in precancerous dysplasia, malignant transformation of hepatocytes, and malignant expansion of tumor cells [52]. According to PPI analysis, ARIDs mostly interact with UBE2D1, HUWE1, FBXO11, and MED23, and these hub genes play essential roles in the tumorigenesis and progression of HCC [53–56]. Several studies focus on the association between hub genes and HCC, however, the exact mechanisms remain to be further studied. Most of the ARID family genes were closely related to immune cells in the HCC microenvironment, suggesting that ARIDs could regulate the immune condition of HCC and may be potential predictors for immune checkpoints during treatment. However, survival analyses in this study were based on public databases. Therefore, more validations are needed to verify our discoveries. Besides, the functional analysis also needs to be verified by *in vitro* and *in vivo* experiments.

## 5. Conclusions

ARIDs mutations, 127 CpGs methylation in all ARIDs, and the expression levels of ARID1A/B, ARID2, ARID3A/B/C, ARID5B, KDM5A/B/C/*D*, and JARID2 were related to the prognosis of patients with HCC. Moreover, ARID3A, KDM5C, and KDM5D were also independent risk factors for the prognostic prediction of HCC. ARIDs may be associated with the regulation of cancer-associated pathways and immune function. Our findings revealed that ARIDs are potential prognostic biomarkers for HCC. ARID family members can be as promising therapeutic targets for HCC. Further research is still needed to validate these results and promote the clinical application of ARID family members in HCC.

## Figures and Tables

**Table 1:** Primer sequences for RT-qPCR.

Gene	Forward (5′-3′)	Reverse (5′-3′)
ARID1A	GCAGCCAAGGAGAGCAGAGTAATC	CTGAGCGAGACTGAGCAACACTG
ARID1B	ATGAACCGCACAGACGATATGATGG	TGGAGGCTGAGGACGACATATAAGG
ARID2	TACTGCCATGTCGTCGTCCTCTAC	GCTGGTGAATGTTGCTGCTGTTG
ARID3B	GACGGAGGTTTGGAAGATGAGGATG	GGTGCTGGAAGTAGATTGGACATGG
ARID3C	GCTCGACCTGTACGCTCTGTTTC	TGATGGTGGTGGGTAGGCTGAG
ARID5A	AGATGATGCCAGGAAAGACCAAAGC	CACAAAGGACACAGAAGACCCAGAG
ARID5B	AGCAAGAAATTCAGGAGGGCAAGG	TCGGTGTGTCTGTAGAGGCTATGG
KDM5A	AATGTGATGGTGGCTGTGATGAGTG	AAGGAAGGAGGTGGTGCTGGAC
KDM5B	CCGCCTCCTAGATTCCAGCAATTC	GTTCTGGCTTCCGTTGTCTCCTC
KDM5C	CCGCCTCCTAGATTCCAGCAATTC	GTTCTGGCTTCCGTTGTCTCCTC
KDM5D	AGAAGCATCCACCAGCCACATTG	TCTCATCCACATCAGCAATCCAAGC
JARID2	CGTCGTGTTCTGTCTGGAGTGTG	ATCGTAGCGGTACATCAACTTCAGC

**Table 2:** Transcriptional levels of AT-rich interaction domains (ARIDs) between HCC and normal liver tissues (ONCOMINE).

No	Gene name	Fold change	*p* value	*T*-test	Datasets
1	ARID1A	1.903	4.62E-6	5.073	Roessler Liver (22)
2	ARID2	1.645	1.29E-6	6.204	Wurmbach Liver (21)
3	ARID3A	2.320	3.60E-12	7.407	Chen Liver (20)
4	ARID4B	1.541	7.11E-11	6.815	Chen Liver (20)
5	ARID4B	2.115	6.32E-9	7.949	Roessler Liver (22)
6	ARID4B	2.135	1.26E-7	6.917	Wurmbach Liver (21)
7	ARID4B	1.871	2.44E-42	15.272	Roessler Liver 2 (22)
8	KDM5A	1.739	7.49E-6	5.075	Wurmbach Liver (21)
9	KDM5B	2.242	1.84E-8	6.837	Wurmbach Liver (21)
10	KDM5B	2.226	4.12E-41	15.473	Roessler Liver 2 (22)
11	KDM5B	1.579	7.87E-8	5.510	Chen Liver (20)
12	KDM5B	1.625	2.20E-5	4.981	Roessler Liver (22)
13	JARID2	1.583	1.57E-35	13.970	Roessler Liver 2 (22)
14	JARID2	1.909	9.63E-5	4.717	Wurmbach Liver (21)

## Data Availability

The datasets analyzed for this study can be found in the TCGA, ONCOMINE, Kaplan–Meier Plotter, MethSurv, cBioPortal, GEPIA, Metascape, and TIMER web resources, and requests to further access to datasets can be directed to 1257003447@qq.com.

## Supplementary Materials

Supplementary Materials Supplementary Figure 1: Spearman correlation of ARID family genes. Supplementary Table 1: the prognostic values of CpGs in the ARID family (MethSurv database, *p* < 0.05). Supplementary Table 2: univariate and multivariate Cox analyses of ARID1A. Supplementary Table 3: univariate and multivariate Cox analyses of ARID1B. Supplementary Table 4: univariate and multivariate Cox analyses of ARID2. Supplementary Table 5: univariate and multivariate Cox analyses of ARID3A. Supplementary Table 6: univariate and multivariate Cox analyses of ARID3B. Supplementary Table 7: univariate and multivariate Cox analyses of ARID3C. Supplementary Table 8: univariate and multivariate Cox analyses of ARID4A. Supplementary Table 9: univariate and multivariate Cox analyses of ARID4B. Supplementary Table 10: univariate and multivariate Cox analyses of ARID5A. Supplementary Table 11: univariate and multivariate Cox analyses of ARID5B. Supplementary Table 12: univariate and multivariate Cox analyses of KDM5A. Supplementary Table 13: univariate and multivariate Cox analyses of KDM5B. Supplementary Table 14: univariate and multivariate Cox analyses of KDM5C. Supplementary Table 15: univariate and multivariate Cox analyses of KDM5D. Supplementary Table 16: nivariate and multivariate Cox analyses of JARID2. Supplementary Table 17: top 10 similar genes of each ARID family member (GEPIA).
