# Supplementary material for: The Prognostic Value of AT-Rich Interaction Domain (ARID) Family Members in Patients with Hepatocellular Carcinoma
Source: Evid Based Complement Alternat Med. 2022 Aug 18;2022:1150390. doi: 10.1155/2022/1150390 (PMC9410793; doi:10.1155/2022/1150390)
Supplement: Supplementary Materials — Supplementary Figure 1: Spearman correlation of ARID family genes. Supplementary Table 1: the prognostic values of CpGs in the ARID family (MethSurv database, p < 0.05). Supplementary Table 2: univariate and multivariate Cox analyses of ARID1A. Supplementary Table 3: univariate and multivariate Cox analyses of ARID1B. Supplementary Table 4: univariate and multivariate Cox analyses of ARID2. Supplementary Table 5: univariate and multivariate Cox analyses of ARID3A. Supplementary Table 6: univariate and multivariate Cox analyses of ARID3B. Supplementary Table 7: univariate and multivariate Cox analyses of ARID3C. Supplementary Table 8: univariate and multivariate Cox analyses of ARID4A. Supplementary Table 9: univariate and multivariate Cox analyses of ARID4B. Supplementary Table 10: univariate and multivariate Cox analyses of ARID5A. Supplementary Table 11: univariate and multivariate Cox analyses of ARID5B. Supplementary Table 12: univariate and multivariate Cox analyses of KDM5A. Supplementary Table 13: univariate and multivariate Cox analyses of KDM5B. Supplementary Table 14: univariate and multivariate Cox analyses of KDM5C. Supplementary Table 15: univariate and multivariate Cox analyses of KDM5D. Supplementary Table 16: nivariate and multivariate Cox analyses of JARID2. Supplementary Table 17: top 10 similar genes of each ARID family member (GEPIA). [file 1150390.f1.zip › Supplementary Tables.docx]

Supplementary Table 1: The prognostic values of CpGs in ARID family (MethSurv database, p < 0.05).

| Gene-CpG | HR | P | Gene-CpG | HR | P |
| --- | --- | --- | --- | --- | --- |
| ARID1A-Body-Open_Sea-cg05300440 | 0.66 | 0.027 | ARID5B-Body-Island-cg00132616 | 1.90 | 0.005 |
| ARID1A-Body-Open_Sea-cg24544309 | 0.67 | 0.042 | ARID5B-Body-Open_Sea-cg02863179 | 0.65 | 0.014 |
| ARID1A-Body-Open_Sea-ch.1.893309F | 0.66 | 0.032 | ARID5B-Body-Island-cg03020000 | 0.64 | 0.020 |
| ARID1B-Body-Open_Sea-cg02247625 | 1.84 | 0.002 | ARID5B-Body-N_Shore-cg03828693 | 0.66 | 0.034 |
| ARID1B-Body-Open_Sea-cg04378942 | 1.91 | 0.000 | ARID5B-Body-Open_Sea-cg03969079 | 1.71 | 0.003 |
| ARID1B-Body-Open_Sea-cg04924555 | 1.82 | 0.012 | ARID5B-TSS200-N_Shore-cg04935939 | 0.48 | 0.000 |
| ARID1B-Body-Open_Sea-cg06012428 | 0.68 | 0.027 | ARID5B-TSS1500-N_Shelf-cg05100313 | 0.61 | 0.006 |
| ARID1B-Body-Open_Sea-cg06019273 | 2.21 | 0.001 | ARID5B-TSS1500-N_Shelf-cg05882781 | 0.45 | 0.000 |
| ARID1B-Body-Open_Sea-cg06108288 | 1.99 | 0.005 | ARID5B-TSS200-N_Shore-cg05976652 | 0.63 | 0.011 |
| ARID1B-3'UTR-Open_Sea-cg07316978 | 2.92 | 0.000 | ARID5B-TSS1500-N_Shore-cg06062519 | 0.41 | 0.000 |
| ARID1B-Body-Open_Sea-cg07965326 | 1.81 | 0.012 | ARID5B-Body-Open_Sea-cg06318796 | 0.61 | 0.012 |
| ARID1B-Body-Open_Sea-cg08268892 | 0.59 | 0.003 | ARID5B-Body-Open_Sea-cg08806401 | 2.71 | 0.000 |
| ARID1B-Body-N_Shelf-cg08783584 | 1.57 | 0.017 | ARID5B-TSS200-N_Shore-cg10708548 | 0.47 | 0.000 |
| ARID1B-Body-Open_Sea-cg10059410 | 1.53 | 0.017 | ARID5B-Body-Open_Sea-cg11283402 | 2.32 | 0.001 |
| ARID1B-Body-Open_Sea-cg10574283 | 0.69 | 0.036 | ARID5B-Body-Open_Sea-cg12616691 | 1.53 | 0.038 |
| ARID1B-TSS1500-Open_Sea-cg10726394 | 1.78 | 0.010 | ARID5B-Body-Open_Sea-cg13958324 | 2.01 | 0.003 |
| ARID1B-Body-Open_Sea-cg10733080 | 2.04 | 0.000 | ARID5B-TSS1500-N_Shelf-cg16013454 | 0.59 | 0.005 |
| ARID1B-Body-Open_Sea-cg11299190 | 2.54 | 0.000 | ARID5B-Body-Open_Sea-cg18547838 | 0.64 | 0.010 |
| ARID1B-Body-Open_Sea-cg12093241 | 0.69 | 0.037 | ARID5B-TSS200-N_Shore-cg19238103 | 0.52 | 0.000 |
| ARID1B-TSS1500-Open_Sea-cg12781778 | 0.60 | 0.004 | ARID5B-Body-Open_Sea-cg22863838 | 1.46 | 0.046 |
| ARID1B-Body-Open_Sea-cg13104508 | 0.68 | 0.028 | ARID5B-Body-Open_Sea-cg24752836 | 2.20 | 0.001 |
| ARID1B-Body-Open_Sea-cg13602547 | 0.62 | 0.013 | ARID5B-TSS1500-N_Shelf-cg26002628 | 0.58 | 0.014 |
| ARID1B-Body-S_Shelf-cg17164954 | 0.69 | 0.035 | ARID5B-Body-Open_Sea-cg27027230 | 2.24 | 0.000 |
| ARID1B-Body-Open_Sea-cg17475813 | 0.64 | 0.019 | KDM5A-Body-N_Shelf-cg12601142 | 1.88 | 0.008 |
| ARID1B-Body-Open_Sea-cg17596249 | 1.79 | 0.015 | KDM5A-3'UTR-Open_Sea-cg20024259 | 2.32 | 0.001 |
| ARID1B-Body-Open_Sea-cg17719099 | 0.65 | 0.018 | KDM5B-Body-Island-cg00571448 | 0.63 | 0.026 |
| ARID1B-Body-Open_Sea-cg20408104 | 1.65 | 0.010 | KDM5B-Body-N_Shore-cg10540110 | 0.61 | 0.008 |
| ARID1B-Body-S_Shore-cg20677939 | 1.60 | 0.045 | KDM5B-TSS200-Island-cg14560895 | 0.67 | 0.025 |
| ARID1B-TSS1500-Open_Sea-cg22432367 | 0.70 | 0.042 | KDM5B-Body-Open_Sea-cg17823829 | 1.96 | 0.003 |
| ARID1B-Body-Open_Sea-cg23303685 | 1.77 | 0.022 | KDM5B-TSS200-Island-cg24354901 | 1.60 | 0.030 |
| ARID1B-Body-Open_Sea-cg23924137 | 1.45 | 0.042 | KDM5C-TSS1500-Island-cg01859586 | 2.08 | 0.002 |
| ARID1B-Body-Open_Sea-cg24962544 | 1.59 | 0.020 | KDM5C-TSS200-Island-cg04927982 | 0.48 | 0.001 |
| ARID1B-Body-Open_Sea-cg26861219 | 1.47 | 0.029 | KDM5C-TSS200-Island-cg12234996 | 0.56 | 0.002 |
| ARID2-3'UTR-Open_Sea-cg00718452 | 2.87 | 0.000 | KDM5C-TSS200-Island-cg16824069 | 0.48 | 0.000 |
| ARID2-Body-Island-cg01183821 | 0.63 | 0.013 | KDM5C-TSS200-Island-cg19903753 | 0.36 | 0.000 |
| ARID2-TSS200-Island-cg01353809 | 0.39 | 0.000 | JARID2-Body-Open_Sea-cg02856481 | 0.64 | 0.011 |
| ARID2-Body-S_Shelf-cg09820373 | 0.56 | 0.008 | JARID2-Body-Open_Sea-cg03129134 | 0.67 | 0.023 |
| ARID2-Body-Open_Sea-cg14659678 | 2.19 | 0.000 | JARID2-TSS1500-Island-cg03223185 | 1.42 | 0.047 |
| ARID2-Body-Open_Sea-cg15085427 | 3.58 | 0.000 | JARID2-Body-Island-cg03721705 | 0.62 | 0.015 |
| ARID2-Body-S_Shore-cg18829183 | 0.65 | 0.018 | JARID2-Body-Open_Sea-cg03846960 | 1.44 | 0.039 |
| ARID2-Body-Open_Sea-cg22526226 | 2.03 | 0.004 | JARID2-Body-Open_Sea-cg04097131 | 1.69 | 0.025 |
| ARID2-Body-Open_Sea-cg23192604 | 2.06 | 0.004 | JARID2-TSS1500-Island-cg06047828 | 1.53 | 0.049 |
| ARID3A-Body-S_Shelf-cg07691306 | 0.60 | 0.006 | JARID2-Body-Open_Sea-cg06382132 | 1.98 | 0.006 |
| ARID3A-TSS200-Island-cg07752304 | 1.46 | 0.030 | JARID2-Body-Open_Sea-cg06607519 | 0.47 | 0.000 |
| ARID3A-TSS200-Island-cg08359958 | 1.97 | 0.003 | JARID2-Body-Open_Sea-cg07532782 | 0.68 | 0.047 |
| ARID3A-3'UTR-Open_Sea-cg15158031 | 1.45 | 0.037 | JARID2-Body-Open_Sea-cg07887224 | 1.55 | 0.014 |
| ARID3A-5'UTR-S_Shore-cg15819921 | 2.01 | 0.003 | JARID2-Body-Open_Sea-cg12582616 | 0.61 | 0.006 |
| ARID3A-Body-Island-cg19546700 | 0.68 | 0.036 | JARID2-Body-Open_Sea-cg13056524 | 1.42 | 0.045 |
| ARID3A-Body-S_Shore-cg22035959 | 0.65 | 0.023 | JARID2-Body-Open_Sea-cg13623749 | 0.63 | 0.015 |
| ARID3A-TSS1500-Island-cg26539232 | 1.76 | 0.014 | JARID2-Body-Open_Sea-cg13877109 | 1.50 | 0.033 |
| ARID3B-Body-Open_Sea-cg00638992 | 1.50 | 0.032 | JARID2-Body-Open_Sea-cg14009473 | 0.68 | 0.037 |
| ARID3B-Body-Open_Sea-cg11077171 | 0.63 | 0.015 | JARID2-Body-Open_Sea-cg14058851 | 0.67 | 0.023 |
| ARID3B-5'UTR-Island-cg19187486 | 0.67 | 0.024 | JARID2-TSS1500-Island-cg14862454 | 1.48 | 0.031 |
| ARID3B-5'UTR;1stExon-Island-cg26705930 | 1.62 | 0.036 | JARID2-TSS1500-Island-cg15109221 | 1.43 | 0.044 |
| ARID3C-TSS1500-S_Shore-cg14466333 | 1.75 | 0.014 | JARID2-Body-Open_Sea-cg16678975 | 1.43 | 0.050 |
| ARID3C-Body-N_Shore-cg21189480 | 1.44 | 0.040 | JARID2-Body-S_Shelf-cg17079858 | 2.06 | 0.002 |
| ARID4A-5'UTR-Island-cg14077301 | 0.64 | 0.017 | JARID2-Body-Open_Sea-cg18569864 | 1.73 | 0.002 |
| ARID4A-5'UTR-S_Shore-cg21184075 | 0.39 | 0.000 | JARID2-Body-Open_Sea-cg18832247 | 0.61 | 0.005 |
| ARID4B-Body-N_Shore-cg00663739 | 0.59 | 0.007 | JARID2-Body-Open_Sea-cg20605134 | 0.68 | 0.027 |
| ARID4B-Body-Open_Sea-cg02985240 | 2.42 | 0.000 | JARID2-Body-S_Shelf-cg22684570 | 1.61 | 0.025 |
| ARID4B-3'UTR-Open_Sea-cg18381955 | 1.70 | 0.007 | JARID2-Body-Open_Sea-cg24408769 | 0.64 | 0.011 |
| ARID4B-Body-Open_Sea-cg24710309 | 1.98 | 0.001 | JARID2-Body-Open_Sea-cg24903767 | 1.62 | 0.035 |
| ARID5A-TSS1500-N_Shore-cg02085953 | 0.63 | 0.018 | JARID2-Body-Open_Sea-cg26405097 | 0.60 | 0.004 |
| ARID5A-5'UTR;1stExon-Island-cg21477691 | 1.64 | 0.022 |  |  |  |

Supplementary Table 2: Univariate and multivariate Cox analyses of ARID1A.

| **Characteristics** | **Univariate analysis** | |  | **Multivariate analysis** | |
| --- | --- | --- | --- | --- | --- |
|  | **Hazard ratio (95% CI)** | **P value** |  | **Hazard ratio (95% CI)** | **P value** |
| Age (>60 vs. <=60) | 1.205 (0.850-1.708) | 0.295 |  |  |  |
| Gender (Male vs. Female) | 0.793 (0.557-1.130) | 0.200 |  |  |  |
| T stage (T3&T4 vs. T1&T2) | 2.598 (1.826-3.697) | **<0.001** |  | 1.650 (0.227-12.010) | 0.621 |
| Pathologic stage (III&IV vs. I&II | 2.504 (1.727-3.631) | **<0.001** |  | 1.535 (0.212-11.098) | 0.671 |
| ARID1A (High vs. Low) | 1.271 (0.899-1.796) | 0.174 |  |  |  |

Supplementary Table 3: Univariate and multivariate Cox analyses of ARID1B.

| **Characteristics** | **Univariate analysis** | |  | **Multivariate analysis** | |
| --- | --- | --- | --- | --- | --- |
|  | **Hazard ratio (95% CI)** | **P value** |  | **Hazard ratio (95% CI)** | **P value** |
| Age (>60 vs. <=60) | 1.205 (0.850-1.708) | 0.295 |  |  |  |
| Gender (Male vs. Female) | 0.793 (0.557-1.130) | 0.200 |  |  |  |
| T stage (T3&T4 vs. T1&T2) | 2.598 (1.826-3.697) | **<0.001** |  | 1.650 (0.227-12.010) | 0.621 |
| Pathologic stage (III&IV vs. I&II | 2.504 (1.727-3.631) | **<0.001** |  | 1.535 (0.212-11.098) | 0.671 |
| ARID1B (High vs. Low) | 1.307 (0.922-1.852) | 0.132 |  |  |  |

Supplementary Table 4: Univariate and multivariate Cox analyses of ARID2.

| **Characteristics** | **Univariate analysis** | |  | **Multivariate analysis** | |
| --- | --- | --- | --- | --- | --- |
|  | **Hazard ratio (95% CI)** | **P value** |  | **Hazard ratio (95% CI)** | **P value** |
| Age (>60 vs. <=60) | 1.205 (0.850-1.708) | 0.295 |  |  |  |
| Gender (Male vs. Female) | 0.793 (0.557-1.130) | 0.200 |  |  |  |
| T stage (T3&T4 vs. T1&T2) | 2.598 (1.826-3.697) | **<0.001** |  | 1.874 (0.256-13.700) | 0.536 |
| Pathologic stage (III&IV vs. I&II | 2.504 (1.727-3.631) | **<0.001** |  | 1.310 (0.180-9.538) | 0.790 |
| ARID2 (High vs. Low) | 1.497 (1.058-2.118) | **0.023** |  | 1.378 (0.951-1.997) | 0.091 |

Supplementary Table 5: Univariate and multivariate Cox analyses of ARID3A.

| **Characteristics** | **Univariate analysis** | |  | **Multivariate analysis** | |
| --- | --- | --- | --- | --- | --- |
|  | **Hazard ratio (95% CI)** | **P value** |  | **Hazard ratio (95% CI)** | **P value** |
| Age (>60 vs. <=60) | 1.205 (0.850-1.708) | 0.295 |  |  |  |
| Gender (Male vs. Female) | 0.793 (0.557-1.130) | 0.200 |  |  |  |
| T stage (T3&T4 vs. T1&T2) | 2.598 (1.826-3.697) | **<0.001** |  | 1.992 (0.272-14.586) | 0.497 |
| Pathologic stage (III&IV vs. I&II | 2.504 (1.727-3.631) | **<0.001** |  | 1.265 (0.174-9.202) | 0.816 |
| ARID3A (High vs. Low) | 1.499 (1.059-2.121) | **0.022** |  | 1.506 (1.041-2.180) | **0.030** |

Supplementary Table 6: Univariate and multivariate Cox analyses of ARID3B.

| **Characteristics** | **Univariate analysis** | |  | **Multivariate analysis** | |
| --- | --- | --- | --- | --- | --- |
|  | **Hazard ratio (95% CI)** | **P value** |  | **Hazard ratio (95% CI)** | **P value** |
| Age (>60 vs. <=60) | 1.205 (0.850-1.708) | 0.295 |  |  |  |
| Gender (Male vs. Female) | 0.793 (0.557-1.130) | 0.200 |  |  |  |
| T stage (T3&T4 vs. T1&T2) | 2.598 (1.826-3.697) | **<0.001** |  | 1.954 (0.267-14.312) | 0.509 |
| Pathologic stage (III&IV vs. I&II | 2.504 (1.727-3.631) | **<0.001** |  | 1.284 (0.176-9.342) | 0.805 |
| ARID3B (High vs. Low) | 1.552 (1.097-2.196) | **0.013** |  | 1.438 (0.995-2.079) | 0.053 |

Supplementary Table 7: Univariate and multivariate Cox analyses of ARID3C.

| **Characteristics** | **Univariate analysis** | |  | **Multivariate analysis** | |
| --- | --- | --- | --- | --- | --- |
|  | **Hazard ratio (95% CI)** | **P value** |  | **Hazard ratio (95% CI)** | **P value** |
| Age (>60 vs. <=60) | 1.205 (0.850-1.708) | 0.295 |  |  |  |
| Gender (Male vs. Female) | 0.793 (0.557-1.130) | 0.200 |  |  |  |
| T stage (T3&T4 vs. T1&T2) | 2.598 (1.826-3.697) | **<0.001** |  | 1.650 (0.227-12.010) | 0.621 |
| Pathologic stage (III&IV vs. I&II | 2.504 (1.727-3.631) | **<0.001** |  | 1.535 (0.212-11.098) | 0.671 |
| ARID3C (High vs. Low) | 0.829 (0.586-1.172) | 0.288 |  |  |  |

Supplementary Table 8: Univariate and multivariate Cox analyses of ARID4A.

| **Characteristics** | **Univariate analysis** | |  | **Multivariate analysis** | |
| --- | --- | --- | --- | --- | --- |
|  | **Hazard ratio (95% CI)** | **P value** |  | **Hazard ratio (95% CI)** | **P value** |
| Age (>60 vs. <=60) | 1.205 (0.850-1.708) | 0.295 |  |  |  |
| Gender (Male vs. Female) | 0.793 (0.557-1.130) | 0.200 |  |  |  |
| T stage (T3&T4 vs. T1&T2) | 2.598 (1.826-3.697) | **<0.001** |  | 1.650 (0.227-12.010) | 0.621 |
| Pathologic stage (III&IV vs. I&II | 2.504 (1.727-3.631) | **<0.001** |  | 1.535 (0.212-11.098) | 0.671 |
| ARID4A (High vs. Low) | 0.914 (0.648-1.289) | 0.608 |  |  |  |

Supplementary Table 9: Univariate and multivariate Cox analyses of ARID4B.

| **Characteristics** | **Univariate analysis** | |  | **Multivariate analysis** | |
| --- | --- | --- | --- | --- | --- |
|  | **Hazard ratio (95% CI)** | **P value** |  | **Hazard ratio (95% CI)** | **P value** |
| Age (>60 vs. <=60) | 1.205 (0.850-1.708) | 0.295 |  |  |  |
| Gender (Male vs. Female) | 0.793 (0.557-1.130) | 0.200 |  |  |  |
| T stage (T3&T4 vs. T1&T2) | 2.598 (1.826-3.697) | **<0.001** |  | 1.650 (0.227-12.010) | 0.621 |
| Pathologic stage (III&IV vs. I&II | 2.504 (1.727-3.631) | **<0.001** |  | 1.535 (0.212-11.098) | 0.671 |
| ARID4B (High vs. Low) | 0.987 (0.696-1.400) | 0.943 |  |  |  |

Supplementary Table 10: Univariate and multivariate Cox analyses of ARID5A.

| **Characteristics** | **Univariate analysis** | |  | **Multivariate analysis** | |
| --- | --- | --- | --- | --- | --- |
|  | **Hazard ratio (95% CI)** | **P value** |  | **Hazard ratio (95% CI)** | **P value** |
| Age (>60 vs. <=60) | 1.205 (0.850-1.708) | 0.295 |  |  |  |
| Gender (Male vs. Female) | 0.793 (0.557-1.130) | 0.200 |  |  |  |
| T stage (T3&T4 vs. T1&T2) | 2.598 (1.826-3.697) | **<0.001** |  | 1.650 (0.227-12.010) | 0.621 |
| Pathologic stage (III&IV vs. I&II | 2.504 (1.727-3.631) | **<0.001** |  | 1.535 (0.212-11.098) | 0.671 |
| ARID5A (High vs. Low) | 0.868 (0.614-1.226) | 0.421 |  |  |  |

Supplementary Table 11: Univariate and multivariate Cox analyses of ARID5B.

| **Characteristics** | **Univariate analysis** | |  | **Multivariate analysis** | |
| --- | --- | --- | --- | --- | --- |
|  | **Hazard ratio (95% CI)** | **P value** |  | **Hazard ratio (95% CI)** | **P value** |
| Age (>60 vs. <=60) | 1.205 (0.850-1.708) | 0.295 |  |  |  |
| Gender (Male vs. Female) | 0.793 (0.557-1.130) | 0.200 |  |  |  |
| T stage (T3&T4 vs. T1&T2) | 2.598 (1.826-3.697) | **<0.001** |  | 1.650 (0.227-12.010) | 0.621 |
| Pathologic stage (III&IV vs. I&II | 2.504 (1.727-3.631) | **<0.001** |  | 1.535 (0.212-11.098) | 0.671 |
| ARID5B (High vs. Low) | 1.248 (0.883-1.762) | 0.209 |  |  |  |

Supplementary Table 12: Univariate and multivariate Cox analyses of KDM5A.

| **Characteristics** | **Univariate analysis** | |  | **Multivariate analysis** | |
| --- | --- | --- | --- | --- | --- |
|  | **Hazard ratio (95% CI)** | **P value** |  | **Hazard ratio (95% CI)** | **P value** |
| Age (>60 vs. <=60) | 1.205 (0.850-1.708) | 0.295 |  |  |  |
| Gender (Male vs. Female) | 0.793 (0.557-1.130) | 0.200 |  |  |  |
| T stage (T3&T4 vs. T1&T2) | 2.598 (1.826-3.697) | **<0.001** |  | 1.650 (0.227-12.010) | 0.621 |
| Pathologic stage (III&IV vs. I&II | 2.504 (1.727-3.631) | **<0.001** |  | 1.535 (0.212-11.098) | 0.671 |
| KDM5A (High vs. Low) | 1.192 (0.844-1.682) | 0.319 |  |  |  |

Supplementary Table 13: Univariate and multivariate Cox analyses of KDM5B.

| **Characteristics** | **Univariate analysis** | |  | **Multivariate analysis** | |
| --- | --- | --- | --- | --- | --- |
|  | **Hazard ratio (95% CI)** | **P value** |  | **Hazard ratio (95% CI)** | **P value** |
| Age (>60 vs. <=60) | 1.205 (0.850-1.708) | 0.295 |  |  |  |
| Gender (Male vs. Female) | 0.793 (0.557-1.130) | 0.200 |  |  |  |
| T stage (T3&T4 vs. T1&T2) | 2.598 (1.826-3.697) | **<0.001** |  | 1.994 (0.272-14.626) | 0.497 |
| Pathologic stage (III&IV vs. I&II | 2.504 (1.727-3.631) | **<0.001** |  | 1.305 (0.180-9.490) | 0.792 |
| KDM5B (High vs. Low) | 1.466 (1.035-2.077) | **0.031** |  | 1.440 (0.992-2.089) | 0.055 |

Supplementary Table 14: Univariate and multivariate Cox analyses of KDM5C.

| **Characteristics** | **Univariate analysis** | |  | **Multivariate analysis** | |
| --- | --- | --- | --- | --- | --- |
|  | **Hazard ratio (95% CI)** | **P value** |  | **Hazard ratio (95% CI)** | **P value** |
| Age (>60 vs. <=60) | 1.205 (0.850-1.708) | 0.295 |  |  |  |
| Gender (Male vs. Female) | 0.793 (0.557-1.130) | 0.200 |  |  |  |
| T stage (T3&T4 vs. T1&T2) | 2.598 (1.826-3.697) | **<0.001** |  | 2.007 (0.274-14.682) | 0.492 |
| Pathologic stage (III&IV vs. I&II | 2.504 (1.727-3.631) | **<0.001** |  | 1.226 (0.168-8.920) | 0.841 |
| KDM5C (High vs. Low) | 1.696 (1.196-2.406) | **0.003** |  | 1.570 (1.083-2.277) | **0.017** |

Supplementary Table 15: Univariate and multivariate Cox analyses of KDM5D.

| **Characteristics** | **Univariate analysis** | |  | **Multivariate analysis** | |
| --- | --- | --- | --- | --- | --- |
|  | **Hazard ratio (95% CI)** | **P value** |  | **Hazard ratio (95% CI)** | **P value** |
| Age (>60 vs. <=60) | 1.205 (0.850-1.708) | 0.295 |  |  |  |
| Gender (Male vs. Female) | 0.793 (0.557-1.130) | 0.200 |  |  |  |
| T stage (T3&T4 vs. T1&T2) | 2.598 (1.826-3.697) | **<0.001** |  | 1.948 (0.267-14.236) | 0.511 |
| Pathologic stage (III&IV vs. I&II | 2.504 (1.727-3.631) | **<0.001** |  | 1.261 (0.173-9.171) | 0.819 |
| KDM5D (High vs. Low) | 0.636 (0.448-0.904) | **0.012** |  | 0.658 (0.452-0.957) | **0.028** |

Supplementary Table 16: Univariate and multivariate Cox analyses of JARID2.

| **Characteristics** | **Univariate analysis** | |  | **Multivariate analysis** | |
| --- | --- | --- | --- | --- | --- |
|  | **Hazard ratio (95% CI)** | **P value** |  | **Hazard ratio (95% CI)** | **P value** |
| Age (>60 vs. <=60) | 1.205 (0.850-1.708) | 0.295 |  |  |  |
| Gender (Male vs. Female) | 0.793 (0.557-1.130) | 0.200 |  |  |  |
| T stage (T3&T4 vs. T1&T2) | 2.598 (1.826-3.697) | **<0.001** |  | 1.650 (0.227-12.010) | 0.621 |
| Pathologic stage (III&IV vs. I&II | 2.504 (1.727-3.631) | **<0.001** |  | 1.535 (0.212-11.098) | 0.671 |
| JARID2-AS1 (High vs. Low) | 0.929 (0.658-1.312) | 0.675 |  |  |  |

Supplementary Table 17: Top 10 similar genes of each ARID family member (GEPIA).

| **ARIDs** | **Similar genes** |
| --- | --- |
| ARID1A | PUM1, SRRM1, HP1BP3, PPP1R8, SETD2, DNAJC14, THRAP3, TNRC18, EP300, RBBP4 |
| ARID1B | SCAF8, LATS1, SHPRH, BCLAF1, MCM9, QKI, MED23, SP3, GOPC, SENP6 |
| ARID2 | SP1, LEMD3, KANSL1, ADNP, ZDHHC17, RIF1, CCNT1, RALGAPB, FBXO11, CSNK2A1 |
| ARID3A | HIC2, DUSP9, TRIM71, IGF2BP1, RP11-923I11.6, BMF, H2AFY2, PNMA3, NR6A1, DNMT3A |
| ARID3B | ZCCHC3, RP5-837I24.1, FAM103A1, EDC3, PLAGL2, FBXL12, CNOT11, HIC2, ARHGAP19, ZWILCH |
| ARID3C | CTD-2529O21.2, SARDH, RP11-84A19.2, TECRL, AL050303.10, AL050303.12, SLC23A3, LA16c-13E4.3, MED15P4, SNORA31 |
| ARID4A | NAA30, GABPA, KLHL28, IREB2, SOS2, PSMA3-AS1, FAM179B, ATAD2B, MORC3, ZNF654 |
| ARID4B | ZC3H11A, RAB3GAP2, SDE2, WDR26, ANGEL2, B3GALNT2, TNKS2, AHCTF1, ACBD3, EXOC8 |
| ARID5A | NAGK, CMTM3, AKNA, DGKZ, BIN1, CD276, GLIPR2, SH3GL1, RHOG, MAP7D1 |
| ARID5B | ZMIZ1, ZNF532, FILIP1L, PDE7A, QTRTD1, CBL, PXDN, CRTC3, SMURF2, UBE2D1 |
| KDM5A | ZNF384, WBP11, PARG, THRAP3, FCF1, ATF1, DNAJC14, ZFR, ARID1A, RNF41 |
| KDM5B | GPATCH2, ZC3H11A, WDR26, ADNP, ZNF765, ZNF124, RAB3GAP2, ARID4B, ADSS, ADNP2 |
| KDM5C | KDM6A, HUWE1, DDX3X, SART3, RALGAPB, MED14, PRDM4, PPP1R12A, EIF1AX, SP1 |
| KDM5D | TXLNGY, RP11-424G14.1, ZFY, UTY, DDX3Y, EIF1AY, USP9Y, NLGN4Y, PRKY, TMSB4Y |
| JARID2 | PBX2, MATR3, SMARCC1, ADNP, CSNK2A1, PRPF4B, BTRC, NUP160, NUP153, LDB1 |
