# Supplementary figures and images for: The Prognostic Value of AT-Rich Interaction Domain (ARID) Family Members in Patients with Hepatocellular Carcinoma
Source: Evid Based Complement Alternat Med. 2022 Aug 18;2022:1150390. doi: 10.1155/2022/1150390 (PMC9410793; doi:10.1155/2022/1150390)

ARID1A

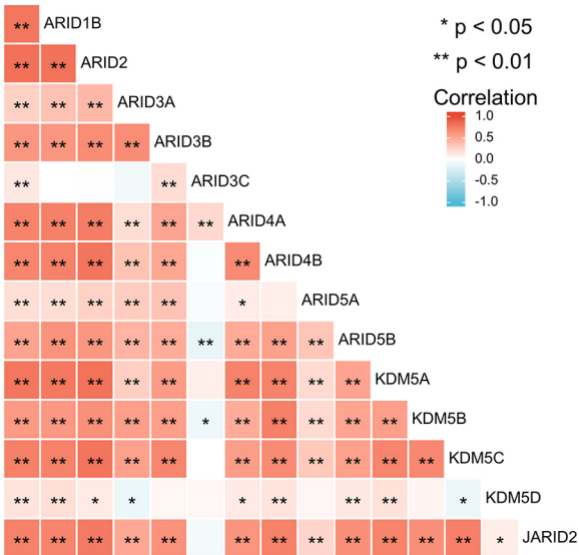

Supplement: Supplementary Materials — Supplementary Figure 1: Spearman correlation of ARID family genes. Supplementary Table 1: the prognostic values of CpGs in the ARID family (MethSurv database, p < 0.05). Supplementary Table 2: univariate and multivariate Cox analyses of ARID1A. Supplementary Table 3: univariate and multivariate Cox analyses of ARID1B. Supplementary Table 4: univariate and multivariate Cox analyses of ARID2. Supplementary Table 5: univariate and multivariate Cox analyses of ARID3A. Supplementary Table 6: univariate and multivariate Cox analyses of ARID3B. Supplementary Table 7: univariate and multivariate Cox analyses of ARID3C. Supplementary Table 8: univariate and multivariate Cox analyses of ARID4A. Supplementary Table 9: univariate and multivariate Cox analyses of ARID4B. Supplementary Table 10: univariate and multivariate Cox analyses of ARID5A. Supplementary Table 11: univariate and multivariate Cox analyses of ARID5B. Supplementary Table 12: univariate and multivariate Cox analyses of KDM5A. Supplementary Table 13: univariate and multivariate Cox analyses of KDM5B. Supplementary Table 14: univariate and multivariate Cox analyses of KDM5C. Supplementary Table 15: univariate and multivariate Cox analyses of KDM5D. Supplementary Table 16: nivariate and multivariate Cox analyses of JARID2. Supplementary Table 17: top 10 similar genes of each ARID family member (GEPIA). [file 1150390.f1.zip › Supplementary Figure 1.pdf]
